# Effect of Polarization Coulomb Field Scattering on Electrical Properties of the 70-nm Gate-Length AlGaN/GaN HEMTs

**DOI:** 10.1038/s41598-018-31313-9

**Published:** 2018-08-27

**Authors:** Peng Cui, Yuanjie Lv, Chen Fu, Huan Liu, Aijie Cheng, Chongbiao Luan, Yang Zhou, Zhaojun Lin

**Affiliations:** 1 School of Microelectronics, Shandong University, Jinan, 250100 China; 2 National Key Laboratory of Application Specific Integrated Circuit (ASIC), Hebei Semiconductor Research Institute, Shijiazhuang, 050051 China; 3 School of Mathematics, Shandong University, Jinan, 250100 China; 4 Key Laboratory of Pulsed Power, Institute of Fluid Physics, CAEP, Mianyang, 621999 China; 5 Microsystem and Terahertz Research Center, China Academy of Engineering Physics, Chengdu, 610200 China

## Abstract

This research presents the first experimental observation of the enhancement of the polarization Coulomb field (PCF) scattering by aggressive lateral scaling of GaN HEMTs. By decreasing the source-drain distance to 300 nm through *n*^+^-GaN ohmic regrowth, 70-nm gate AlGaN/GaN HEMTs achieved an extremely low electron mobility. Different from the electron mobility of the traditional device, which was determined by polar optical phonon scattering, the electron mobility of the 70-nm gate AlGaN/GaN HEMTs was dominated by PCF scattering due to the enhanced nonuniform strain distribution of the AlGaN barrier layer. Furthermore, compared with the parasitic access resistance at gate-source voltage *V*_GS_ = 0 V, the parasitic access resistance at *V*_GS_ = −2.5 V showed an increase of approximately 700%, which was also responsible for the enhanced PCF scattering.

## Introduction

AlGaN/GaN high electron mobility transistors (HEMTs) have shown excellent performance in the RF domain due to their high electron velocities and large sheet electron densities^1–6^. In RF applications, the cutoff frequency (*f*_T_), maximum oscillation frequency (*f*_max_), and maximum drain current are the key device performance parameters^1,7–9^. Many innovative device scaling technologies have been presented^10–16^, and device scaling successfully increases the *f*_T_ and *f*_max_ of GaN HEMTs^17–20^. However, because of the piezoelectric and spontaneous polarization, there are polarization charges at the AlGaN/GaN interface^21–24^. Under the gate bias, the polarization charges of the AlGaN barrier layer under the gate region change due to the converse piezoelectric effect, which causes the strain distribution of the AlGaN barrier layer to be altered^25–27^. The strain distribution variation is more obvious with device scaling; as a result, the strain-distribution-dependent polarization Coulomb field scattering has a stronger influence on the electron transport^28–35^, and the RF application of AlGaN/GaN HEMTs is affected. Therefore, it is essential to investigate this influence to further improve the RF device performance of AlGaN/GaN HEMTs.

In this research, 70-nm gate AlGaN/GaN HEMTs with different source-drain distances (*L*_SD_ = 300/600 nm) and gate widths (*W*_G_ = 20/40 μm) were fabricated. Based on the measured current-voltage characteristics and the obtained two-dimensional electron gas (2DEG) electron densities, the electron mobility and parasitic access resistances were determined, and the influence of the strain distribution on the 70-nm gate AlGaN/GaN HEMTs was explored.

## Results and Discussion

**Figure 1:**
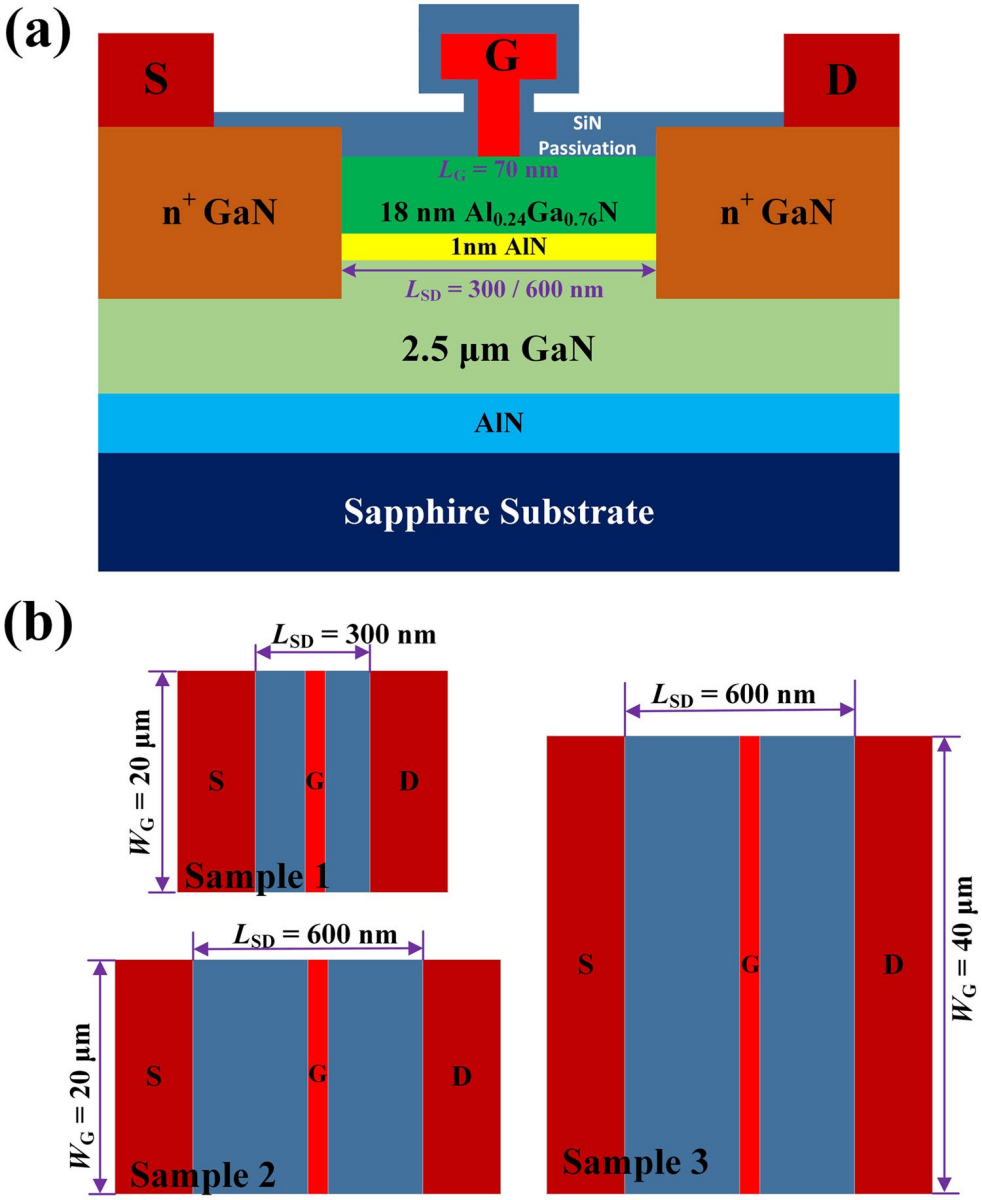
(**a**) Schematic of the AlGaN/GaN HEMTs. (**b**) Top views of the three samples.

**Figure 2:**
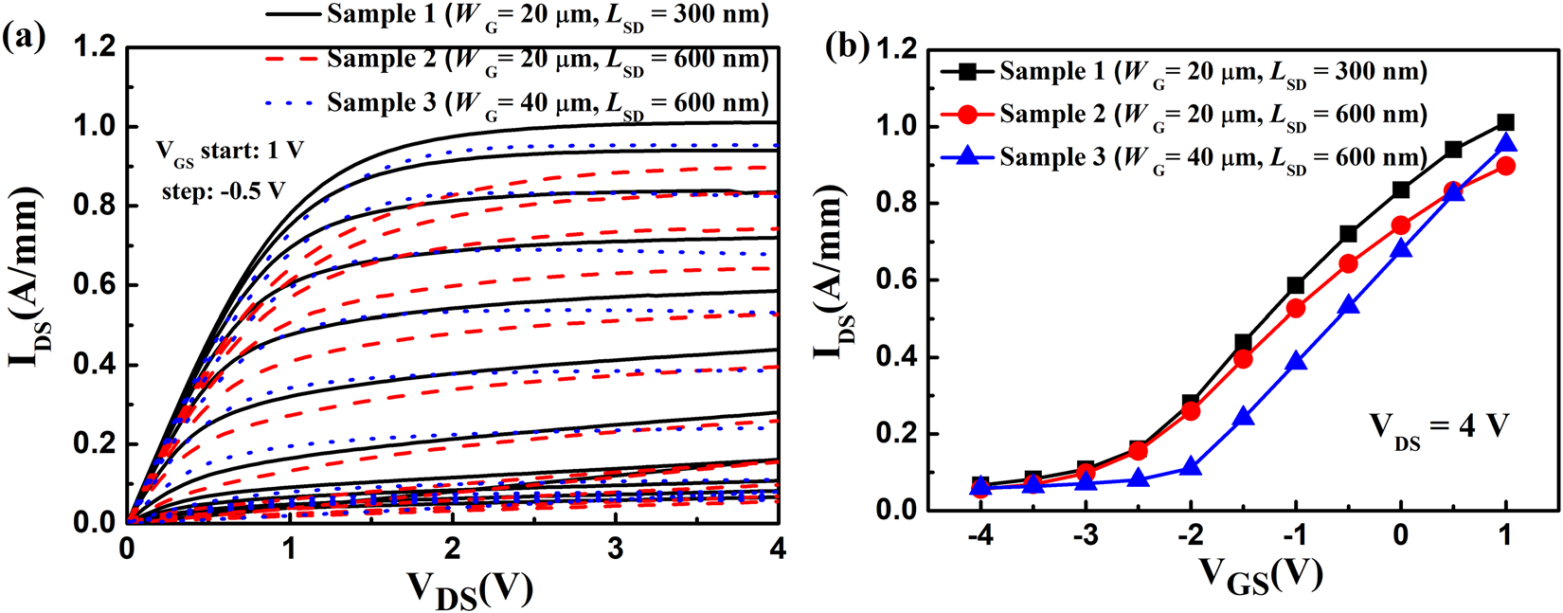
The measured (**a**) *I*–*V* out characteristics and (**b**) transfer characteristics (at drain-source voltage *V*_DS_ = 4 V) of the three samples.

**Figure 3:**
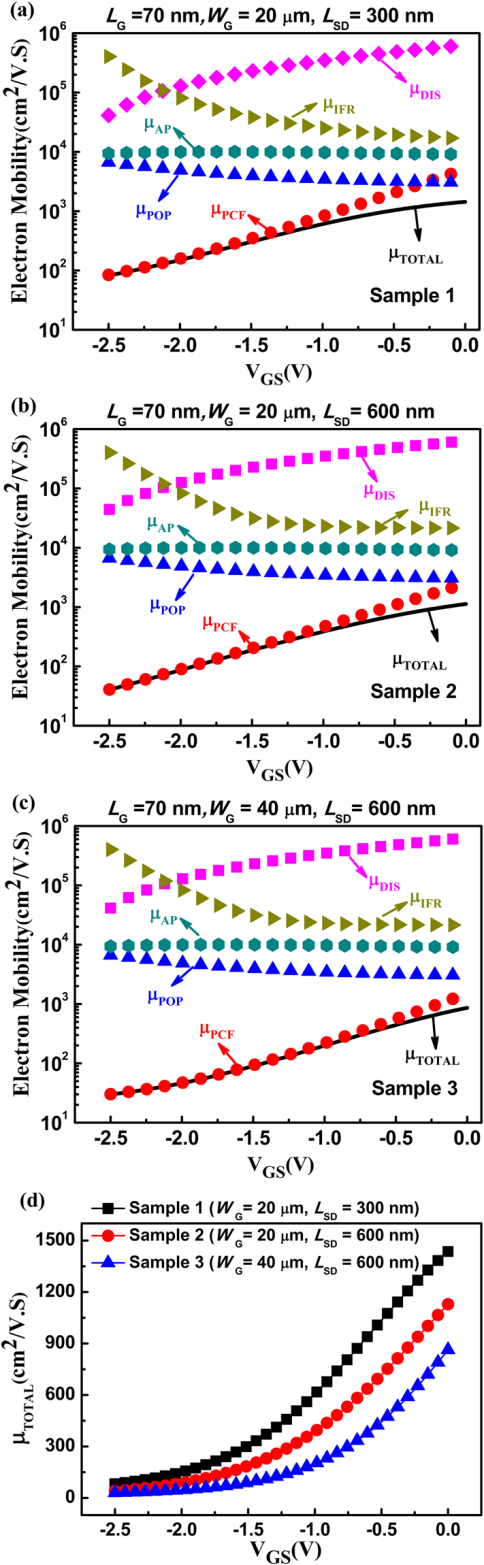
The detailed electron mobility as determined by PCF (μ_PCF_), POP (μ_POP_), IFR (μ_IFR_), AP (μ_AP_), and DIS (μ_DIS_) scatterings, and the total electron mobility (μ _TOTAL_) as a function of the gate-source voltage for (**a**) Sample 1, (**b**) Sample 2, and (**c**) Sample 3, respectively. (**d**) The total electron mobility (μ _TOTAL_) versus the gate-source voltage for the three samples.

The schematic of the AlGaN/GaN HEMTs were shown in Fig. 1(a). As shown in Fig. 1(b), the device with *L*_SD_ = 300 nm and *W*_G_ = 20 μm was labeled as Sample 1, the device with *L*_SD_ = 600 nm and *W*_G_ = 20 μm as Sample 2, the device with *L*_SD_ = 600 nm and *W*_G_ = 40 μm as Sample 3. The *I*–*V* output characteristics and transfer characteristics (at drain-source voltage *V*_DS_ = 4 V) of the three samples were measured, as shown in Fig. 2. For decreasing the influence of the short-channel effect, the values of the measured drain-source current *I*_DS_ with a drain-source voltage *V*_DS_ of 100 mV at different gate biases were used. The main scattering mechanisms in the AlGaN/GaN HEMTs include polar optical phonon (POP), polarization Coulomb field (PCF), interface roughness (IFR), acoustic phonon (AP), and dislocation (DIS) scatterings^25,28,31,36–41^. Based on the two-dimensional scattering theory, the 2DEG electron mobility was obtained by applying the self-consistent iteration calculation^25,26,42^, as shown in Fig. 3. The detailed parameters and calculation process are the same as in ref.^42^. As the gate-source voltage was decreased from 0 V to −2.5 V, the 2DEG electron mobility of the three samples decreased. The detailed electron mobility determined by POP, IFR, AP, and DIS scatterings (labeled as μ_POP_, μ_IFR_, μ_AP_, and μ_DIS_, respectively) was the same for the three samples^25,26^. However, the electron mobility determined by PCF scattering, labeled as μ_PCF_, differed among the three samples. The PCF scattering in the 70-nm gate AlGaN/GaN HEMTs was the strongest scattering mechanism and obviously dominated the total electron mobility variation, causing the electron mobility to decrease with the gate bias. The electron mobility of Sample 2 was lower than that of Sample 1 but higher than that of Sample 3. This means that an increase in the source-drain distance or gate width can decrease the electron mobility.

**Figure 4:**
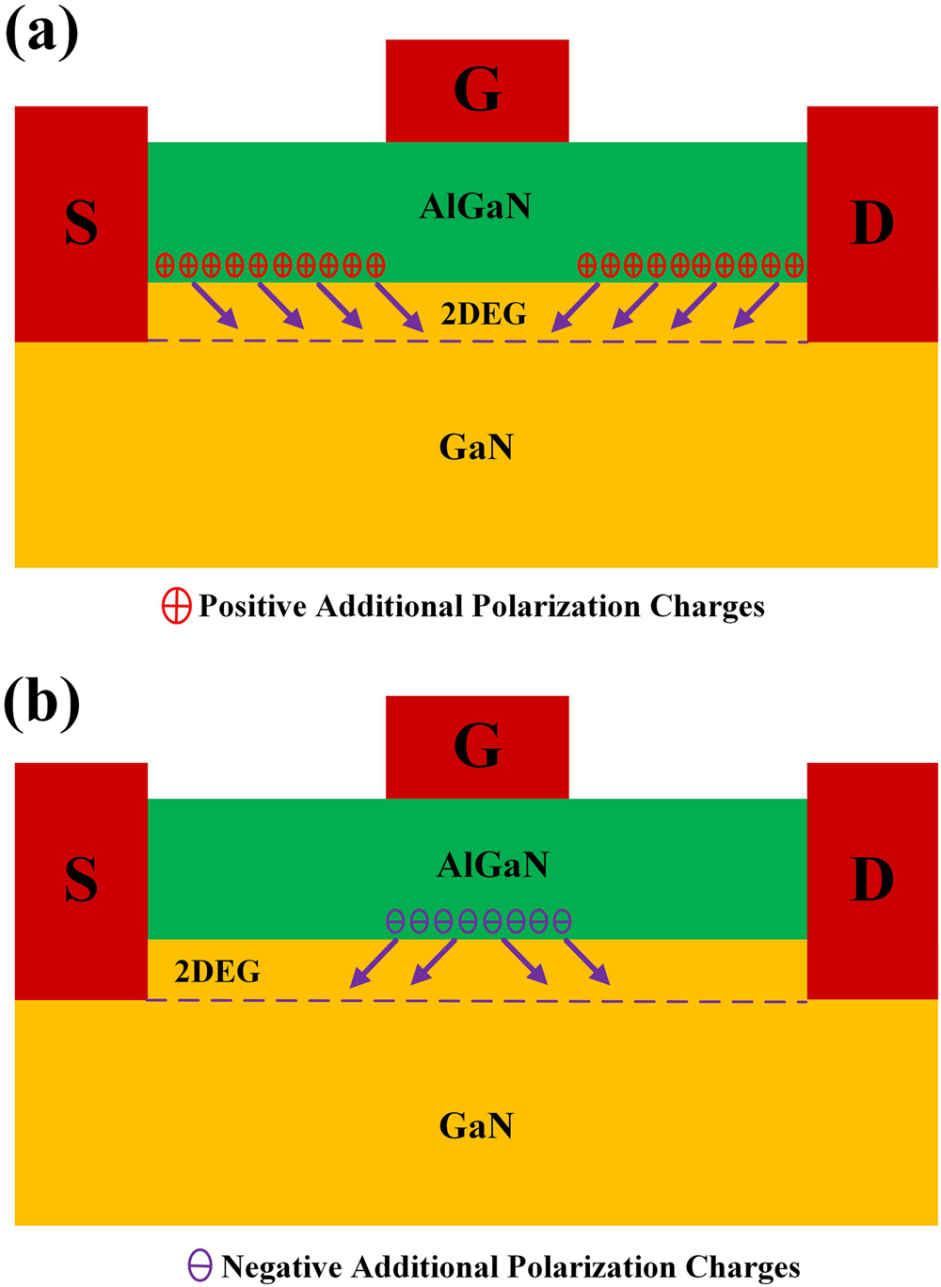
Schematic of the influence of the additional polarization charges on the 2DEG electrons (**a**) under the gate region and (**b**) under the gate-source/gate-drain region.

Before the device processing, the strain distribution in the AlGaN barrier layer was consistent, and the polarization charges at the AlGaN/GaN interface were uniform^21–24^. Because of the converse piezoelectric effect, the gate bias can change the strain of the AlGaN barrier layer under the gate region, causing the strain distribution of the AlGaN barrier layer to become nonuniform^25–27^. The device scaling makes the nonuniformly distributed strain more obvious. This means that the non-uniform distribution of the polarization charges is enhanced. The PCF scattering, which originates from the nonuniform distribution of the polarization charges at the AlGaN/GaN interface, is also enhanced with the device scaling^25,26,28,31–33^. The difference between the non-uniformly distributed polarization charges and the uniformly distributed ones is defined as the additional polarization charges. The negative gate bias decreases the tensile strain of the AlGaN barrier layer and reduces the polarization charges under the gate region^25–27^. When the effect of the PCF scattering on the electron under the gate region is considered, as shown in Fig. 4(a), the positive additional polarization charges are located at the gate-source and gate-drain regions, which are increased with the decrease of the gate bias^25–27^. The PCF scattering is enhanced by the increased additional polarization charges^25–27,31–33^, causing the electron mobility to decrease with the decreased gate bias. Samples 1 and 2 have the same gate length and width. However, Sample 2 has bigger gate-source and gate-drain regions, as well as a larger number of additional polarization charges, compared with Sample 1. This means that the PCF scattering of Sample 2 is stronger than that of Sample 1. Therefore, Sample 2 has lower electron mobility. Samples 2 and 3 have the same gate length, gate-source spacing, and gate-drain spacing. Because Sample 3 has a bigger gate width, the increased additional polarization charges originating from this increased gate width enhances the PCF scattering^34,35^; thus, Sample 3 has lower electron mobility than Sample 2.

**Figure 5:**
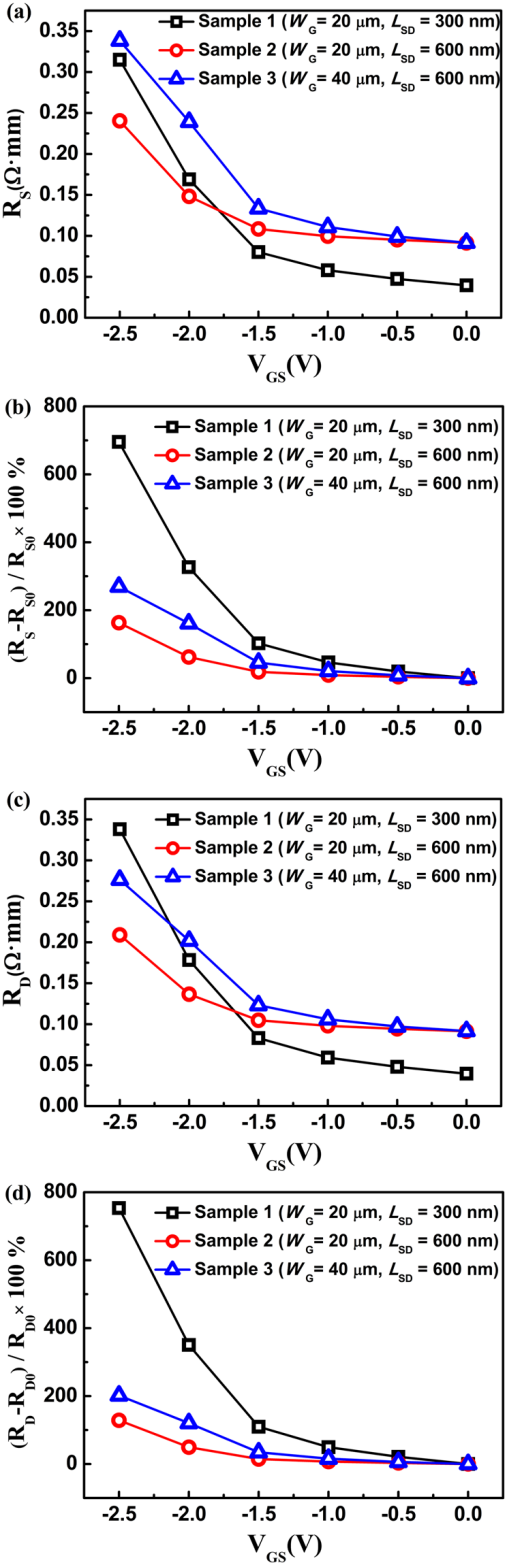
The obtained (**a**) source and (**c**) drain parasitic access resistance versus the gate-source voltage. The percentage variation of the (**b**) source and (**d**) drain parasitic access resistance corresponding to the source/drain access resistance at gate-source voltage *V*_GS_ = 0 V.

Figure 5(a and c) shows the calculated parasitic access resistance versus the gate-source voltage. The gate-source parasitic access resistance (*R*_S_) and the gate-drain parasitic access resistance (*R*_D_) are obviously increased with the decreased gate-source bias. The difference between the parasitic access resistance under the negative gate bias (*R*_S_ and *R*_D_) and that under the zero gate bias (*R*_S0_ and *R*_D0_) was also obtained, as shown in Fig. 5(b and d). Compared with the parasitic access resistance at gate-source voltage *V*_GS_ = 0 V, the parasitic access resistance at *V*_GS_ = −2.5 V showed an increase of approximately 700%. Furthermore, Samples 1 and 3 both showed a bigger increase than Sample 2. This means that the device with a smaller gate-source/gate-drain distance or a larger gate width has a more obvious increase in parasitic access resistance with the decreased gate-source bias.

Under the negative gate-source voltage, the polarization charges under the gate region are lower than those under the gate-source/gate-drain region. Therefore, considering the electrons in the gate-source/gate-drain channel, as shown in Fig. 4(b), the negative additional polarization charges were under the gate region, and increased with the decreased gate-source bias^24,25^. The increased additional polarization charges enhanced the PCF scattering and increased the parasitic access resistance. The additional polarization charges under the gate region are the same for Samples 1 and 2. However, Sample 1 has smaller gate-source/gate-drain distances than Sample 2. Thus, the influence of the additional polarization charges on the smaller gate-source/gate-drain distances is stronger^24,25^, causing the PCF scattering to be enhanced and making the increase in the parasitic access resistances more obvious. Sample 3 has a bigger gate width, and the additional polarization charges under the gate region are larger for Sample 3 than for Sample 2. Furthermore, the PCF scattering is stronger and the parasitic access resistances showed a larger increase for Sample 3^34,35^.

## Conclusions

In summary, the electron mobility and parasitic access resistances versus the gate-source voltage for the 70-nm gate AlGaN/GaN HEMTs were obtained. The PCF scattering, originating from the strain variation of the AlGaN barrier layer, was shown to have a more significant influence on the device characterization with device scaling. This could present a possible approach toward improving the performance of 70-nm gate AlGaN/GaN HEMTs by decreasing the PCF scattering.

## Methods

### Sample fabrication

The AlGaN/GaN heterostructures were grown on a sapphire substrate by molecular beam epitaxy (MBE) (see Fig. 1(a) for a more detailed material structure description). The sheet electron concentration and electron mobility obtained from Hall measurements were 9.27 × 10^12^ cm^−2^ and 2020 cm^2^/V•s, respectively. The AlGaN barrier layer in the ohmic contact regions was etched into the GaN channel layer by inductively coupled plasma reactive ion etching (ICP-RIE), followed by MOCVD re-growth of highly Si doped *n*^+^-GaN (3 × 10^19^ cm^−3^). The source and drain electrodes were formed by using non-alloyed Ti/Pt. The transmission-line matrix measurements showed that the ohmic contact resistance *R*_C_ was 0.58 Ω•mm. Ni/Au T-shaped gate with a 70-nm gate length (*L*_G_) was fabricated and located in the middle of the source and drain contacts. Finally, the devices were passivated by using 50-nm-thick SiN deposited by PECVD. As shown in Fig. 1(b), the device with *L*_SD_ = 300 nm and *W*_G_ = 20 μm was labeled as Sample 1, the device with *L*_SD_ = 600 nm and *W*_G_ = 20 μm as Sample 2, the device with *L*_SD_ = 600 nm and *W*_G_ = 40 μm as Sample 3.

### Measurements

The current-voltage (*I*–*V*) measurements were carried out at room temperature by using an Agilent B1500A semiconductor parameter analyzer.
